# Associations between Repeated Measures of Maternal Urinary Phthalate Metabolites and Thyroid Hormone Parameters during Pregnancy

**DOI:** 10.1289/EHP170

**Published:** 2016-05-06

**Authors:** Lauren E. Johns, Kelly K. Ferguson, Thomas F. McElrath, Bhramar Mukherjee, John D. Meeker

**Affiliations:** 1 Department of Environmental Health Sciences, University of Michigan School of Public Health, Ann Arbor, Michigan, USA; 2 Division of Maternal and Fetal Medicine, Brigham and Women’s Hospital, Harvard Medical School, Boston, Massachusetts, USA; 3 Department of Biostatistics, University of Michigan School of Public Health, Ann Arbor, Michigan, USA

## Abstract

**Background:**

Maintaining thyroid homeostasis during pregnancy is essential for normal fetal growth and development. Growing evidence suggests that phthalates interfere with normal thyroid function. Few human studies have investigated the degree to which phthalates may affect thyroid hormone levels in particularly susceptible populations such as pregnant women.

**Objectives:**

We examined the associations between repeated measures of urinary phthalate metabolites and plasma thyroid hormone levels in samples collected at up to four time points per subject in pregnancy. Additionally, we investigated the potential windows of susceptibility to thyroid hormone disturbances related to study visit of sample collection.

**Methods:**

Data were obtained from pregnant women (n = 439) participating in a nested case–control study of preterm birth with 116 cases and 323 controls. We measured 9 phthalate metabolite concentrations in urine samples collected at up to four study visits per subject during pregnancy (median = 10, 18, 26, and 35 weeks of gestation, respectively). We also measured a panel of thyroid function markers in plasma collected at the same four time points per subject during pregnancy.

**Results:**

Although our results were generally null, in repeated measures analyses we observed that phthalate metabolites were largely inversely associated with thyrotropin and positively associated with free and total thyroid hormones. Cross-sectional analyses by study visit revealed that the magnitude and/or direction of these relationships varied by timing of exposure during gestation.

**Conclusions:**

These results support previous reports showing the potential for environmental phthalate exposure to alter circulating levels of thyroid hormones in pregnant women.

**Citation:**

Johns LE, Ferguson KK, McElrath TF, Mukherjee B, Meeker JD. 2016. Associations between repeated measures of maternal urinary phthalate metabolites and thyroid hormone parameters during pregnancy. Environ Health Perspect 124:1808–1815; http://dx.doi.org/10.1289/EHP170

## Introduction

Maintaining thyroid homeostasis during pregnancy is essential for normal fetal growth and development, and especially for early fetal neurodevelopment (Hartoft-Nielsen et al. 2011; Préau et al. 2015; Williams 2008). Human health studies have shown that both overt and subclinical maternal thyroid disease (hyper- and hypothyroidism) may be associated with adverse birth outcomes such as preterm birth (Aggarawal et al. 2014; Casey et al. 2005; Su et al. 2011), low birth weight (Aggarawal et al. 2014; Chen et al. 2014; Millar et al. 1994; Phoojaroenchanachai et al. 2001), and impaired fetal growth (Aggarawal et al. 2014; Chen et al. 2014; Saki et al. 2014), although similar associations have not been observed for maternal subclinical hyperthyroidism (Casey et al. 2006). Notably, these birth outcomes are associated with lasting physical and neurodevelopmental complications among surviving infants (IOM et al. 2007).

Phthalate diesters have been commonly used as plasticizers and solvents in a variety of consumer and industrial products (ATSDR 2001, 2002). Because of their extensive use, phthalate metabolites have been consistently detected in humans, and more specifically in pregnant women worldwide (Adibi et al. 2003; Cantonwine et al. 2014; Casas et al. 2011; Meeker et al. 2009). Growing scientific evidence suggests that this group of environmental chemicals may interfere with normal thyroid function (Boas et al. 2009; Kashiwagi et al. 2009).

Animal and *in vitro* studies suggest that phthalates may be capable of disrupting circulating thyroid hormone levels, although the exact biological mechanism(s) of action remain unclear (Boas et al. 2012; Liu et al. 2015; Zhai et al. 2014). Additionally, a limited number of epidemiological studies have shown that phthalates may alter thyroid hormone levels in adult men and nonpregnant women as well as children (Boas et al. 2010; Meeker et al. 2007; Meeker and Ferguson 2011). Less is known about the degree to which phthalates may affect thyroid function in other vulnerable populations such as pregnant women.

To date, three epidemiological investigations have assessed the relationships between phthalate exposure and thyroid hormone levels in pregnant women (Huang et al. 2007; Johns et al. 2015; Kuo et al. 2015). Although the findings reported in these investigations provide suggestive evidence for the potential thyroid-disrupting effects of phthalates during pregnancy, these studies are limited by study design and/or sample size. The present analyses build upon this existing research on the possible role of phthalates in disturbing thyroid hormone levels in pregnant women by investigating similar associations in a large nested case–control study. Here, we examined the associations between repeated measures of urinary phthalate metabolites and plasma thyroid hormone levels in samples collected at up to four time points per subject in pregnancy. Additionally, we investigated the potential windows of susceptibility to phthalate exposure related to study visit of sample collection.

## Methods

### Study Population

This was a secondary analysis of data from a nested case–control study with the primary aim of investigating the effects of environmental phthalate exposure on the risk of preterm birth (Ferguson et al. 2014a). The study population includes a subset of pregnant women participating in the ongoing LifeCodes prospective birth cohort. All pregnant women who planned to deliver at the Brigham and Women’s Hospital in Boston, Massachusetts, who were > 18 years old, and whose initial visit was before 15 weeks of gestation were eligible to participate and were recruited between 2006 and 2008. The only exclusion criterion was higher-order multiple gestations (e.g., triplets or greater) (McElrath et al. 2012). Additional information regarding recruitment as well as sample collection and processing are described in detail elsewhere (Ferguson et al. 2014a, 2014b; McElrath et al. 2012). Briefly, at the initial study visit (median, 9.71 weeks gestation; range, 4.71–19.1 weeks), participants completed a questionnaire to collect sociodemographic information (e.g., race/ethnicity, income, health insurance provider) and relevant health information (e.g., tobacco and alcohol use, family health history), and provided urine and blood samples for biomarker analysis. Participants were followed until delivery, and provided relevant health information [e.g., body mass index (BMI) and blood pressure] as well as urine and blood samples at three additional study visits: visit 2 (median, 17.9 weeks gestation; range, 14.9–32.1 weeks), visit 3 (median, 26.0 weeks gestation; range, 22.9–36.3 weeks), and visit 4 (median, 35.1 weeks gestation; range, 33.1–38.3 weeks).

Approximately 1,600 women were enrolled in the original cohort at the Brigham and Women’s Hospital, and 1,181 were followed until delivery and had a singleton live birth. In 2011, 130 women who delivered a preterm singleton infant (< 37 completed weeks of gestation) and 352 randomly selected women who delivered singletons at or after 37 weeks of gestation were included in the nested case–control study. In the current analysis, we additionally excluded women diagnosed with thyroid disease based on medical records (e.g., diagnosed hyper- or hypothyroidism, Grave’s disease, or thyroid cancer) (*n* = 41) and those who did not provide blood samples at any study visit during follow-up (*n* = 2). The final study population (*n* = 439) included 116 preterm birth cases and 323 controls. The study protocols were approved by the ethics and research committees of the participating institutions and all study participants gave written informed consent.

### Thyroid Hormone Measurements

We assayed plasma samples (*n* = 439 participants; *n* = 1,445 total samples) collected up to four time points in pregnancy at the Clinical Ligand Assay Service Satellite (CLASS) Lab at the University of Michigan (Ann Arbor, MI). Samples were analyzed for thyrotropin (or thyroid-stimulating hormone; TSH) as well as total triiodothyronine (T_3_) and thyroxine (T_4_) using an automated chemiluminescence immunoassay according to manufacturer’s instructions (Bayer ADVIA Centaur; Siemens Health Care Diagnostics, Inc.). We measured free T_4_ using direct equilibrium dialysis followed by radioimmunoassay (IVD Technologies). The manufacturer did not provide trimester-specific reference ranges for TSH. In their absence, the American Thyroid Association recommends the following for TSH: first trimester, 0.1–2.5 μIU/mL; second trimester, 0.2–3.0 μIU/mL; third trimester, 0.3–3.0 μIU/mL (Stagnaro-Green et al. 2011). The free T_4_ pregnancy reference ranges provided by the laboratory were as follows: first trimester, 0.7–2.0 ng/dL; second trimester, 0.5–1.6 ng/dL; third trimester, 0.5–1.6 ng/dL. The limits of detection (LOD) were 0.01 μIU/mL for TSH, 10 ng/dL for total T_3_, 0.3 μg/dL for total T_4_, and 0.1 ng/dL for free T_4_. Thyroid hormone concentrations less than the LOD were assigned a value of LOD divided by the square root of 2 (Hornung and Reed 1990).

In addition to exploring individual thyroid hormone parameters, we calculated the ratio of T_3_ to T_4_ (T_3_/T_4_) from the respective total hormone concentrations. The T_3_/T_4_ ratio is an index of thyroid homeostasis and reflects the action of thyroid hormones on peripheral tissues (Dietrich et al. 2012; Mortoglou and Candiloros 2004).

### Phthalate Metabolite Measurements

NSF International (Ann Arbor, MI) analyzed available urine samples (*n* = 439 participants; *n* = 1,443 samples), also collected up to four times in pregnancy, for phthalate metabolites using a method developed by the Centers for Disease Control and Prevention (CDC) described elsewhere (Lewis et al. 2013; Silva et al. 2007). Briefly, the analytical technique involved enzymatic deconjugation of metabolites from their glucuronidated form, solid-phase extraction, separation by high-performance liquid chromatography, and detection by tandem mass spectrometry. The following nine metabolites were measured in urine samples: mono(2-ethylhexyl) phthalate (MEHP), mono-*n*-butyl phthalate (MBP), mono(2-ethyl-5-hydroxyhexyl) phthalate (MEHHP), mono(2-ethyl-5-oxohexyl) phthalate (MEOHP), mono(2-ethyl-5-carboxypentyl) phthalate (MECPP), mono-benzyl phthalate (MBzP), mono-iso-butyl phthalate (MiBP), mono-ethyl phthalate (MEP), and mono(3-carboxypropyl) phthalate (MCPP). LODs for individual metabolites were in the low microgram per liter range (Ferguson et al. 2014b). As with the hormones, phthalate metabolite concentrations below the LOD were assigned a value of LOD divided by the square root of 2 (Hornung and Reed 1990). In addition to examining individual phthalate metabolites, we created a variable for the molar sum (μmol/L) of the four measured di(2-ethylhexyl) phthalate (DEHP) metabolites (MEHP, MEHHP, MEOHP, and MECPP; ΣDEHP) (Meeker et al. 2009).

To correct for urinary dilution in univariate analyses, we standardized phthalate metabolite concentrations using specific gravity (SG) according to the following equation: *PSG* = *P*[(1.015 – 1)/(*SG* – 1)], where *PSG* is the specific gravity–adjusted phthalate metabolite concentration (μg/L), *P* is the observed phthalate metabolite concentration, 1.015 is the specific gravity population median, and *SG* is the specific gravity of the urine sample (Meeker et al. 2009). Unadjusted phthalate metabolite concentrations were used in multivariate analyses with SG added as a separate covariate, because modeling corrected metabolite levels may introduce bias (Barr et al. 2005).

### Statistical Analyses

To make our study population more representative of the original cohort from which the case–control sample arose, we applied inverse probability weighting to all analyses considering association between secondary variables measured under case–control sampling. Specifically, we corrected for over-representation of preterm birth cases by applying study-specific weights related to the inverse probability of inclusion of controls so that the relative weights of cases and controls in the present study population were similar to what would be observed in the overall LifeCodes cohort (Richardson et al. 2007).

The empirical histogram of total T_3_ as well as free and total T_4_ approximately resembled a normal distribution. The distributions of TSH as well as all nine phthalate metabolites and ΣDEHP were right-skewed; thus, we used the natural log transformation of these variables for statistical analyses. We tabulated means and percentiles for all urinary phthalate metabolites and plasma thyroid hormones. We calculated geometric means and geometric standard deviations for log-normally distributed variables. We examined the distribution of thyroid hormone parameters by study visit of sample collection and demographic characteristics. We calculated Spearman correlations between phthalate metabolites using SG-corrected values. We used linear mixed models (LMMs) with subject-specific random intercepts and slopes for gestational age at sample collection to test the differences in repeated measures of thyroid hormone levels by each categorical covariate that were introduced as predictors in the mixed-model regression.

In repeated measures analyses, we explored the associations between urinary phthalate metabolites and plasma thyroid hormone concentrations across pregnancy using LMMs with one hormone regressed on one phthalate metabolite per model, with each model including a subject-specific random intercept and slope for gestational age at sample collection. Crude models included fixed-effects terms for gestational age at sample collection and urinary SG. Full models were additionally adjusted for maternal age at enrollment, BMI at time of sample collection, and health insurance provider. We chose maternal age and BMI *a priori* as covariates in full models because of their known associations with thyroid hormone concentrations and urinary phthalate metabolite levels (Hatch et al. 2008; Iacobellis et al. 2005; Peeters 2008). We identified additional covariates based on ≥ 10% change in the main effect estimates when added to the models.

In our secondary analyses, we investigated the cross-sectional relationships between urinary phthalate metabolites and plasma thyroid hormone concentrations at each study visit (visits 1–4) using linear regression models with one phthalate metabolite and one outcome variable per model. We adjusted these models for maternal age at enrollment, BMI at time of sample collection, health insurance provider, and urinary specific gravity. To enhance the interpretation of statistical models containing log-transformed exposure and/or outcome variables, we expressed all regression coefficients and associated 95% confidence intervals (CIs) as the percent change in thyroid hormone levels for an interquartile range (IQR) increase in urinary phthalate metabolite concentrations. We considered associations statistically significant at the 0.05 level. We performed all data analyses using SAS version 9.3 (SAS Institute Inc.).

## Results

Population characteristics of the case–control study population as well as the distributions of the phthalate metabolites by study visit have been previously reported (Ferguson et al. 2014a, 2015). Bivariate analyses showed that thyroid hormone concentrations significantly varied by certain demographic characteristics (Table 1). Specifically, TSH concentrations were significantly lower among pregnant women who identified as African-American or other race/ethnicity compared to white, and who had public health insurance compared to private. Women who reported no alcohol use during pregnancy had higher concentrations of TSH than did those who reported drinking alcohol. For free T_4_, concentrations were significantly lower among women who graduated from technical school than among those with a high school diploma or the equivalent, and lower among women who were obese (> 30 kg/m^2^) than among those with a BMI < 25 kg/m^2^.

**Table 1:** Thyroid hormone measurements [weighted median (25th, 75th percentiles)] by demographic characteristics in all samples measured (*n *= 439 participants, 1,443 plasma samples).

Population characteristics	Percent of total population^*a*^	TSH (μIU/mL)	Free T_4_ (ng/dL)	Total T_3_ (ng/dL)	Total T_4_ (μg/dL)	T_3_/T_4_ ratio^*b*^
Age (years)
18–24 (reference)	13	1.04 (0.70, 1.60)	1.12 (0.91, 1.36)	179 (155, 209)	11.2 (10.1, 12.3)	16.2 (13.6, 18.8)
25–29	21	1.20 (0.79, 1.64)	1.13 (0.87, 1.35)	157 (130, 186)*	10.4 (9.30, 11.6)	15.1 (13.2, 17.4)*
30–34	40	1.25 (0.81, 1.75)	1.09 (0.86, 1.35)	149 (127, 182)*	10.0 (8.90, 11.3)*	14.8 (12.7, 17.6)*
≥ 35	26	1.33 (0.96, 1.83)	1.11 (0.87, 1.37)	149 (124, 184)*	10.0 (8.90, 11.2)*	14.8 (12.5, 17.7)*
Race/ethnicity
White (reference)	56	1.36 (0.97, 1.88)	1.09 (0.86, 1.34)	148 (127, 182)	10.0 (8.90, 11.1)	14.8 (12.6, 17.6)
African American	17	0.96 (0.70, 1.35)*	1.11 (0.90, 1.32)	169 (145, 198)*	10.9 (9.60, 12.4)*	15.5 (13.6, 17.9)*
Other	27	1.12 (0.70, 1.66)*	1.16 (0.88, 1.41)	162 (130, 192)*	10.4 (9.20, 11.9)*	15.4 (12.8, 18.2)
Education
High school (reference)	15	1.13 (0.69, 1.60)	1.14 (0.93, 1.43)	171 (147, 200)	11.1 (9.90, 12.3)	15.6 (13.3, 18.4)
Technical school	17	1.09 (0.76, 1.64)	1.09 (0.86, 1.31)*	164 (136, 195)	10.3 (9.00, 11.6)*	16.2 (13.6, 18.6)
Junior college or some college	29	1.30 (0.90, 1.82)	1.10 (0.84, 1.35)	152 (132, 183)*	10.0 (8.80, 11.3)*	14.9 (13.0, 17.6)*
College graduate	39	1.30 (0.89, 1.83)	1.09 (0.87, 1.37)	147 (124, 178)*	10.1 (9.10, 11.2)*	14.6 (12.5, 17.0)*
Health insurance provider
Private/HMO/Self-pay (reference)	80	1.27 (0.85, 1.79)	1.10 (0.87, 1.35)	150 (127, 182)	10.0 (8.90, 11.3)	14.8 (12.7, 17.6)
Medicaid/SSI/MassHealth	20	1.06 (0.72, 1.60)*	1.13 (0.90, 1.36)	182 (153, 214)*	11.1 (10.0, 12.4)*	16.2 (13.8, 18.6)*
BMI at initial visit (kg/m^2^)
< 25 (reference)	53	1.25 (0.82, 1.79)	1.15 (0.89, 1.42)	144 (122, 169)	10.1 (9.00, 11.4)	14.0 (12.0, 16.5)
25–30	26	1.28 (0.84, 1.78)	1.11 (0.87, 1.35)	168 (142, 194)*	10.4 (9.30, 11.6)	16.1 (13.8, 18.7)*
> 30	21	1.17 (0.78, 1.66)	1.05 (0.83, 1.25)*	181 (142, 208)*	10.3 (8.90, 11.6)	17.1 (14.3, 19.6)*
Tobacco use
Smoked during pregnancy (reference)	7	1.23 (0.85, 1.60)	1.13 (0.85, 1.35)	171 (145, 209)	10.2 (9.10, 11.1)	16.8 (13.9, 20.7)
No smoking during pregnancy	93	1.25 (0.81, 1.76)	1.10 (0.87, 1.35)	154 (129, 185)*	10.3 (9.10, 11.6)	15.0 (12.8, 17.6)*
Alcohol use
Alcohol use during pregnancy (reference)	5	0.93 (0.66, 1.34)	1.10 (0.96, 1.34)	154 (118, 182)	9.60 (8.10, 10.8)	16.5 (14.0, 19.4)
No alcohol use during pregnancy	95	1.25 (0.82, 1.76)*	1.11 (0.87, 1.35)	156 (130, 186)	10.3 (9.10, 11.6)*	15.0 (12.9, 17.7)
Fetal sex
Male (reference)	46	1.28 (0.85, 1.73)	1.09 (0.88, 1.33)	157 (130, 187)	10.3 (9.00, 11.6)	15.4 (13.3, 18.0)
Female	54	1.22 (0.80, 1.77)	1.12 (0.89, 1.38)	154 (129, 185)	10.2 (9.10, 11.5)	14.7 (12.5, 17.6)
Abbreviations: BMI, body mass index; HMO, Health Maintenance Organization; SSI, Supplemental Security Income. ^***a***^Weighted by case–control sampling probabilities to represent the general sampling population. ^***b***^Total T_3_ expressed in ng/dL and total T_4_ in μg/dL. *Significant difference (*p* < 0.05) in thyroid hormone concentration in the category compared with reference (first category listed) using linear mixed models with a random intercept and slope for each subject.						

All thyroid hormone parameters were detected in most samples in this study population (precent detected for total T_4_ and T_3_ = 100%, TSH = 99.5%, and free T_4_ = 98%), and measurable concentrations of the nine urinary phthalate metabolites were detected in at least 95% of urine samples (Ferguson et al. 2014a, 2015). Correlations between phthalate metabolites were strongest for DEHP metabolites (Spearman *r* = 0.74–0.93), were moderate between MBzP, MBP, and MiBP (*r* = 0.43–0.61) and between DEHP metabolites and MCPP (*r* = 0.35–0.44), and were weak between all other metabolites (*r* = –0.03 to 0.29). Weighted geometric mean concentrations of urinary and plasma biomarkers varied by study visit of sample collection (Table 2). Compared with visit 1, we observed significantly decreased levels of all DEHP metabolites and MCPP at visit 3. We detected significantly increased concentrations of MBzP, MBP, MiBP, and MEP at visit 4. For the hormones, compared with visit 1, we found significantly increased levels of TSH at visits 2–4, whereas free T_4_ levels were significantly lower at these three subsequent study visits.

**Table 2:** Weighted distributions of urinary and plasma biomarkers by study visit of sample collection in pregnancy (*n *= 439 subjects).

Biomarker	Samples (*n*)^*a*^	Geometric mean (geometric standard deviation)			
		Visit 1 (median, 10 weeks gestation)	Visit 2 (median, 18 weeks gestation)	Visit 3 (median, 26 weeks gestation)	Visit 4 (median, 35 weeks gestation)
Phthalate metabolites^*b*^
MEHP (μg/L)	1,541	10.6 (3.52)	10.9 (3.39)	9.46 (3.28)*	9.83 (3.52)*
MEHHP (μg/L)	1,541	34.7 (3.37)	34.8 (3.10)	27.2 (3.21)*	36.6 (3.33)
MEOHP (μg/L)	1,541	18.6 (3.28)	18.3 (3.03)	15.6 (3.19)*	20.9 (3.22)
MECPP (μg/L)	1,541	44.4 (3.35)	42.6 (3.25)*	36.8 (3.31)*	49.3 (3.35)
ΣDEHP (μmol/L)	1,541	0.39 (3.16)	0.38 (3.01)	0.32 (3.04)*	0.42 (3.18)
MBzP (μg/L)	1,541	7.36 (3.07)	7.34 (3.15)	7.05 (2.93)	8.03 (2.94)*
MBP (μg/L)	1,541	18.3 (2.39)	18.4 (2.53)	17.3 (2.50)	19.7 (2.11)*
MiBP (μg/L)	1,541	7.66 (2.29)	7.14 (2.38)	7.45 (2.32)	9.05 (2.17)*
MEP (μg/L)	1,541	145 (4.66)	144 (4.84)	141 (4.48)	156 (4.99)*
MCPP (μg/L)	1,541	2.11 (3.09)	2.25 (3.26)*	1.94 (2.89)*	2.04 (2.77)
Thyroid hormones
TSH (μIU/mL)	1,210	1.13 (2.11)	1.30 (1.90)*	1.26 (1.67)*	1.31 (1.71)*
Free T_4_ (ng/dL)^*c*^	1,435	1.49 (0.87)	1.16 (0.63)*	1.08 (0.81)*	0.99 (0.49)*
Total T_3_ (ng/dL)^*c*^	1,130	140 (39.9)	166 (38.9)*	170 (39.8)*	171 (41.6)*
Total T_4_ (μg/dL)^*c*^	1,391	10.2 (2.03)	10.7 (1.73)*	10.5 (1.97)*	10.2 (2.04)
T_3_/T_4_ ratio^*c*^	1,120	13.8 (2.67)	15.5 (3.43)*	16.5 (4.00)*	17.1 (4.46)*
^***a***^Number of plasma samples per hormone varied due to limitations in sample volume. ^***b***^Urinary phthalate concentrations corrected for specific gravity. ^***c***^Arithmetic mean and standard deviation reported. *Significant difference (*p* < 0.05) in urinary phthalate metabolite concentration or thyroid hormone compared to visit 1 (reference) using linear mixed models with a random intercept for each subject.					

Associations from repeated measures analyses using fully adjusted LMMs were similar to those observed in crude unadjusted models (data not shown). We detected a significant inverse relationship between MEHP and TSH, where an IQR increase in MEHP was associated with a 5.31% (95% CI: –10.1, –0.23) decrease in TSH (Table 3). We also observed significant inverse associations between MiBP [percent change in outcome for an IQR increase in exposure (%Δ) = –9.51; 95% CI: –16.4, –2.01] and MCPP (%Δ = –6.63; 95% CI: –11.6, –1.41). We detected generally positive associations between each metabolite and free T_4_, with a significant relationship observed for MCPP (%Δ = 6.91; 95% CI: 1.70, 12.1). Finally, we observed significant positive associations between MEP and both total T_3_ (%Δ = 2.24; 95% CI: 0.32, 4.17) and the T_3_/T_4_ ratio (%Δ = 2.87; 95% CI: 1.27, 4.47) as well as between MEHP and total T_4_ (%Δ = 1.29; 95% CI: 0.26, 2.32).

**Table 3:** Repeated measures analysis: percent change (95% CIs) in thyroid hormone concentrations in relation to interquartile range increase in urinary phthalate metabolite concentrations.

Analyte	ln-TSH		Free T_4_		Total T_3_		Total T_4_		T_3_/T_4_ ratio	
	%Δ (95% CI)	*p*-Value	%Δ (95% CI)	*p*-Value	%Δ (95% CI)	*p*-Value	%Δ (95% CI)	*p*-Value	%Δ (95% CI)	*p*-Value
MEHP	–5.31 (–10.1, –0.23)	0.04*	4.15 (–0.87, 9.16)	0.10	0.28 (–1.29, 1.85)	0.72	1.29 (0.26, 2.32)	0.01*	–1.05 (–2.33, 0.23)	0.11
MEHHP	–3.95 (–8.67, 1.01)	0.11	2.67 (–2.27, 7.62)	0.29	0.97 (–0.55, 2.50)	0.21	0.66 (–0.34, 1.66)	0.19	–0.06 (–1.31, 1.19)	0.93
MEOHP	–3.74 (–8.38, 1.15)	0.13	3.89 (–0.99, 8.77)	0.12	1.08 (–0.41, 2.58)	0.16	0.86 (–0.13, 1.84)	0.09	–0.15 (–1.38, 1.08)	0.81
MECPP	–3.98 (–9.17, 1.51)	0.15	4.89 (–0.52, 10.3)	0.08	0.86 (–0.83, 2.54)	0.32	0.86 (–0.25, 1.97)	0.13	–0.23 (–1.61, 1.15)	0.74
ΣDEHP	–4.33 (–9.23, 0.84)	0.10	4.09 (–1.12, 9.29)	0.12	0.82 (–0.77, 2.41)	0.31	0.87 (–0.17, 1.91)	0.10	–0.29 (–1.59, 1.01)	0.66
MBzP	–4.5 (–11.26, 2.78)	0.22	2.57 (–3.89, 9.03)	0.43	0.47 (–1.79, 2.74)	0.68	1.04 (–0.47, 2.55)	0.18	–0.60 (–2.47, 1.26)	0.52
MBP	–2.66 (–8.95, 4.07)	0.43	2.83 (–3.37, 9.04)	0.37	1.10 (–0.92, 3.13)	0.29	0.24 (–1.14, 1.62)	0.73	0.85 (–0.82, 2.53)	0.32
MiBP	–9.51 (–16.4, –2.01)	0.01*	3.61 (–3.48, 10.7)	0.32	0.99 (–1.51, 3.49)	0.44	0.47 (–1.23, 2.17)	0.56	0.54 (–1.58, 2.66)	0.62
MEP	–4.56 (–10.4, 1.70)	0.15	–0.48 (–6.33, 5.38)	0.87	2.24 (0.32, 4.17)	0.02*	–0.48 (–1.79, 0.82)	0.47	2.87 (1.27, 4.47)	0.00*
MCPP	–6.63 (–11.6, –1.41)	0.01*	6.91 (1.70, 12.1)	0.01*	1.55 (–0.11, 3.21)	0.07	0.14 (–0.96, 1.23)	0.81	1.30 (–0.05, 2.64)	0.06
Linear mixed models include random intercept and slope for each subject and were adjusted for urinary specific gravity, gestational age at time of sample collection, maternal age at enrollment, body mass index (BMI) at time of sample collection, and health insurance provider. *p < 0.05.										

**Figure 1:**
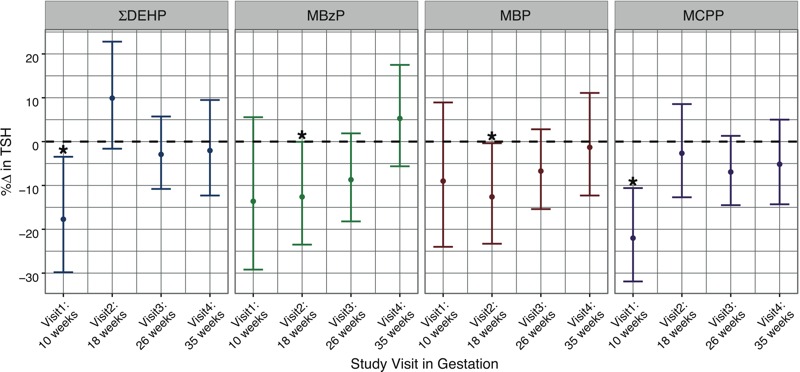
Cross-sectional analysis: percent change in TSH concentrations in relation to an interquartile range increase in urinary phthalate metabolite concentrations (*p < 0.05).

**Figure 2:**
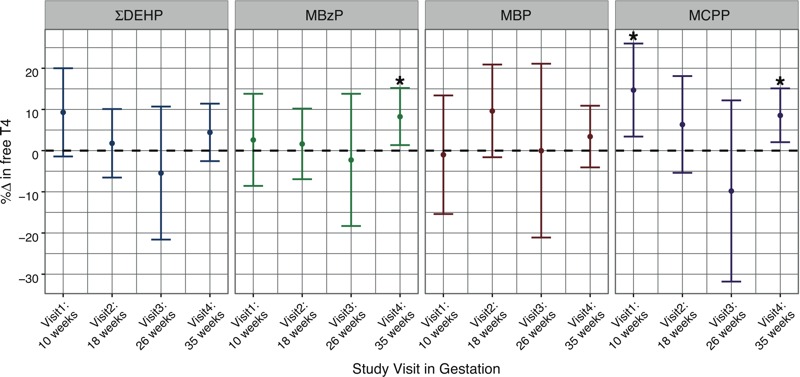
Cross-sectional analysis: percent change in free T_4_ concentrations in relation to an interquartile range increase in urinary phthalate metabolite concentrations (*p < 0.05).

To explore potential windows of susceptibility, we stratified linear regression analyses by time of sample collection in pregnancy (see Table S1). Similar to the results of repeated measures analyses, phthalate metabolites were generally inversely associated with TSH at each of the four study visits, although statistically significant associations were observed only in visits 1 and 2 (Figure 1). We detected significant positive associations between several urinary phthalate metabolites and free T_4_ at visits 1 and 4. In contrast to repeated measures analyses, phthalate metabolites were inversely related to free T_4_ at visit 3, although these associations were not statistically significant (Figure 2). For total hormones, ΣDEHP was significantly positively associated with T_3_ and T_4_ at visit 1, and with T_4_ at visit 4. We observed null associations between metabolites and the T_3_/T_4_ ratio at visits 1–3, with inverse associations for ΣDEHP, including several individual DEHP metabolites, at visit 4.

## Discussion

In the largest cohort study conducted on this topic to date, we report significant associations between several phthalate metabolites and thyroid hormone parameters in samples collected at up to four time points in pregnancy. In repeated measures analyses, we observed that phthalate metabolites were largely inversely associated with TSH and positively associated with free and total thyroid hormones. Cross-sectional analyses by study visit revealed that the magnitude and/or direction of these relationships varied by time point of exposure during gestation. We detected inverse relationships between several metabolites (particularly DEHP metabolites) and TSH at visits 1 and 2, whereas no significant associations were observed in the latter half of pregnancy. For free T_4_, we observed generally positive associations at all study visits except for visit 3 (median, 26 weeks of gestation), where these associations became inverse in direction. These results suggest that environmental phthalate exposure may alter thyroid hormone parameters in pregnant women. Moreover, our findings indicate that the timing of phthalate exposure during gestation may be important for a pregnant woman’s susceptibility to thyroidal disruption.

Three epidemiological studies have previously investigated the potential phthalate-associated alterations in thyroid hormone parameters among pregnant women (Huang et al. 2007; Johns et al. 2015; Kuo et al. 2015). Notably, only one of these investigations, which we conducted using pilot data, assessed the relationships using biomarker measurements collected at multiple time points in pregnancy (Johns et al. 2015). In that population of pregnant women in Puerto Rico, which used data from two study visits in pregnancy, no statistically significant associations were observed between urinary phthalate metabolites and serum concentrations of TSH or free T_4_ in repeated measures analyses. However, in cross-sectional analyses, we previously observed a significant positive association between MiBP and free T_4_ at a median of 18 weeks gestation as well as inverse associations between several phthalate metabolites, including ΣDEHP, and free T_4_ at a median of 26 weeks gestation (Johns et al. 2015). Although these associations were similar in direction to the corresponding results reported at visit 2 (median, 18 weeks of gestation) and visit 3 (median, 26 weeks of gestation) in the present study, here we did not report statistically significant associations for free T_4_ at either visit. The discrepant results observed between these two studies may be attributable to differences in: population size, number of serial biological samples available as well as the timing of sample collection in pregnancy, phthalate exposure levels, laboratory methods used to measure free hormones, and/or population demographic characteristics.

Our results for free and total T_4_ also differ from those reported in a prior cross-sectional study conducted among a cohort of Taiwanese pregnant women undergoing amniocentesis (*n* = 76) (Huang et al. 2007). Huang et al. (2007) observed significant inverse associations between urinary MBP and both plasma free and total T_4_ at mean 27.9 weeks of gestation. In contrast, we observed largely null and in some cases positive associations between phthalate metabolites, including MBP, and free or total T_4_ in both repeated measures and cross-sectional analyses. In a more recent cross-sectional analysis conducted among a separate cohort of Taiwanese pregnant women (*n* = 148), Kuo et al. (2015) observed significant inverse unadjusted associations between several urinary phthalate metabolites (MEOHP, MEHHP, and MBzP) and serum TSH in the third trimester. Likewise, we generally found inverse relationships between phthalate metabolites and TSH in both repeated measures and cross-sectional analyses, although these were specific to visits early in gestation.

The pattern of results reported in the present study, specifically those observed for urinary DEHP metabolites, conflict with findings from previous human health studies conducted among adult men and nonpregnant women. In a cross-sectional study of men recruited from a fertility clinic, urinary concentrations of MEHP were inversely associated with free T_4_ and total T_3_ (Meeker et al. 2007). Urinary concentrations of DEHP metabolites were also inversely associated with total T_3_ and total and free T_4_ in a representative sample of U.S. adults (Meeker and Ferguson 2011). No significant associations were observed for TSH in either study. It is possible that differences in exposure levels and/or in the physiological state of participants (i.e., pregnancy) may have contributed to discrepancies in the results between these studies and the present study.

Various biological mechanisms have been proposed through which phthalates may act to alter thyroid function. Phthalates may exert thyroid-disrupting action at multiple points along the hypothalamic–pituitary–thyroid axis. It has been suggested that phthalates may bind to thyroid hormone receptors and alter their signaling, although evidence for overt binding is lacking (Kashiwagi et al. 2009; Zoeller 2005, 2007). Additionally, limited *in vitro* studies have shown that phthalates may have thyroid hormone receptor antagonist activity (Shen et al. 2009; Sugiyama et al. 2005). Several studies have also demonstrated potential phthalate actions on thyroid hormone biosynthesis and biotransport (Breous et al. 2005; Ishihara et al. 2003; Liu et al. 2015; Wenzel et al. 2005; Zhai et al. 2014).

Phthalates may also affect the peripheral metabolism of thyroid hormones. To our knowledge, this is the first study to investigate the effects of phthalates on thyroid homeostasis using the T_3_/T_4_ ratio. This ratio has been used as an index of the peripheral conversion of T_4_ to T_3_ (the more biologically active hormone) by deiodinase enzymes, and can be high or low in certain thyroid disease states (Dietrich et al. 2012; Mortoglou and Candiloros 2004). Here, we observed a statistically significant positive association between urinary MEP and the T_3_/T_4_ ratio in repeated measures analyses. Cross-sectional analyses by study visit revealed significant inverse associations with several phthalate metabolites, including DEHP metabolites, at visit 4. Although we did not directly measure deiodinase activity in tissues, these results suggest that phthalates may influence circulating levels of thyroid hormones in pregnant women by altering the peripheral metabolism of thyroid hormones. Indeed, limited animal studies have shown that certain phthalates and/or their metabolites may influence the gene expression of deiodinase enzymes (Liu et al. 2015; Zhai et al. 2014). However, additional research is required to examine the influences of phthalates on extrathyroidal regulation of thyroid hormone production in humans, particularly in tissues relevant to pregnancy (e.g., the placenta).

Because each organ system develops at different time points in pregnancy and because any disturbances in the normal growth and maturation of these systems may have lasting consequences on the developing fetus, the health effects of *in utero* exposures depend not only on the structure and dose of the chemical but also on the timing of exposure in gestation (Schug et al. 2011). In humans, the fetus relies exclusively on maternal thyroid hormones in the first trimester until the fetal thyroid gland becomes fully functional after 18 weeks of gestation (Glinoer et al. 1990; Obregon et al. 2007). In later pregnancy, maternal thyroid hormones are essential for fetal thyroid homeostasis (Hartoft-Nielsen et al. 2011). Even mild alterations in circulating thyroid hormones in pregnancy may have important implications for fetal health. In pregnant women with normal range free T_4_ and TSH levels, increases in free T_4_ in the first trimester were associated with lower birth weight and an increased risk of small for gestational age (Medici et al. 2013). Notably, we observed significant phthalate-associated increases in free T_4_ levels at study visit 1 (first trimester) in the present study.

Our study was limited by the lack of iodine status of our study participants, which is a trace element essential for normal thyroid function (Zimmermann and Köhrle 2002). Although recent population-based studies have shown that pregnant women in the United States may have less than adequate median urinary iodine levels (Caldwell et al. 2013), it is unlikely that this would be a confounder in the phthalate–thyroid hormone associations. Although some studies have observed correlations between urinary iodine and phthalate concentrations, it is unclear whether an individual’s phthalate exposure directly influences iodine status or whether both are simply found in the same dietary source. Moreover, in a study conducted among a representative sample of U.S. adult men and women, iodine excretion had a negligible impact on the significant relationships observed between phthalate metabolites and thyroid hormone levels (Mendez and Eftim 2012). An additional limitation is that we did not assess the thyroid autoimmunity of the study participants. It is possible that the associations observed in our study may differ by level of anti-thyroid antibodies, which may be present in approximately 10–20% of pregnant women (Stagnaro-Green et al. 1990; Wang et al. 2011). Finally, we performed a number of comparisons, and there is the potential that some of the observed associations may have been attributable to chance. We did not correct for multiple comparisons because available methods (e.g., Bonferroni adjustments) are often too conservative due to underlying assumptions of independence and increase the probability of type 2 errors, thereby potentially masking truly important differences (Perneger 1998). Despite these limitations, our study has many strengths. We have investigated the effects of environmental phthalate exposure on maternal thyroid hormone levels in the largest longitudinal study to date. The collection of biomarker measurements at multiple time points in pregnancy allows for the use of statistical modeling techniques to more powerfully detect associations among repeated measurements. Furthermore, our analytical method for measuring free T_4_ is advantageous over traditional immunoassays because it is specific and not influenced by serum binding proteins, which change dramatically over normal pregnancy (Lee et al. 2009; Nelson et al. 1994).

## Conclusions

Overall, the results from our analyses support previous reports showing the potential for environmental phthalate exposure to disturb circulating levels of thyroid hormones in pregnant women. Additional human health and animal studies are required to resolve the direction of the specific relationships, to further elucidate periods of vulnerability in pregnancy to phthalate exposure, and to reveal the specific biological mechanisms involved at phthalate levels comparable with those to which humans (and more specifically, pregnant women) are environmentally exposed. Furthermore, the implications of these findings to maternal and fetal health need to be determined.

## Supplemental Material

(154 KB) PDF
