# Supplementary material for: Associations between Repeated Measures of Maternal Urinary Phthalate Metabolites and Thyroid Hormone Parameters during Pregnancy
Source: Environ Health Perspect. 2016 May 6;124(11):1808–15. doi: 10.1289/EHP170 (PMC5089879; doi:10.1289/EHP170)
Supplement: (154 KB) PDF [file EHP170.s001.acco.pdf]

Environ Health Perspect
DOI: 10.1289/EHP170

Note to readers with disabilities: *EHP* strives to ensure that all journal content is accessible to all readers. However, some figures and Supplemental Material published in *EHP* articles may not conform to 508 standards due to the complexity of the information being presented. If you need assistance accessing journal content, please contact ehp508@niehs.nih.gov. Our staff will work with you to assess and meet your accessibility needs within 3 working days.

Supplemental Material

Associations between Repeated Measures of Maternal Urinary Phthalate Metabolites and Thyroid Hormone Parameters during Pregnancy

Lauren E. Johns[1], Kelly K. Ferguson[1], Thomas F. McElrath[2], Bhramar Mukherjee, and John D. Meeker[1]

Table of Contents

Table S1: Cross-sectional analysis: percent change (95% CIs) in thyroid hormone concentrations in relation to interquartile range increase in urinary phthalate metabolite concentrations by study visit during gestation

Table S1. Cross-sectional analysis: percent change (95% CIs) in thyroid hormone concentrations in relation to interquartile range increase in urinary phthalate metabolite concentrations by study visit during gestation

Analyte	%Δ (95%CI)	p-value	%Δ (95%CI)	p-value	%Δ (95%CI)	p-value	%Δ (95%CI)	p-value	%Δ (95%CI)	p-value
**Visit 1 : median 10 weeks of gestation**										
	**ln-TSH**		**Free T4**		**Total T3**		**Total T4**		**T3/T4 Ratio**	
MEHP	-16.0 (-27.6, -2.55)	0.02*	8.24 (-1.06, 17.5)	0.08	-0.11 (-3.98, 3.77)	0.96	0.20 (-2.45, 2.85)	0.88	-0.26 (-2.89, 2.36)	0.84
MEHHP	-14.9 (-26.1, -1.90)	0.03*	3.22 (-6.00, 12.4)	0.49	0.76 (-3.22, 4.74)	0.71	0.04 (-2.72, 2.80)	0.98	1.00 (-1.70, 3.71)	0.46
MEOHP	-15.7 (-27.5, -2.01)	0.03*	5.84 (-2.94, 14.6)	0.19	1.19 (-2.62, 4.99)	0.54	0.42 (-2.21, 3.06)	0.75	0.87 (-1.72, 3.46)	0.51
MECPP	-17.8 (-30.6, -2.52)	0.02*	10.5 (0.86, 20.1)	0.03*	0.57 (-3.55, 4.70)	0.78	0.67 (-2.12, 3.45)	0.64	0.13 (-2.66, 2.91)	0.93
ΣDEHP	-17.7 (-29.8, -3.46)	0.02*	9.30 (-1.42, 20.0)	0.09	0.68 (-3.87, 5.23)	0.77	0.51 (-2.61, 3.62)	0.75	0.44 (-2.64, 3.52)	0.78
MBzP	-13.6 (-29.2, 5.57)	0.15	2.60 (-8.56, 13.8)	0.65	-0.14 (-4.94, 4.66)	0.95	0.62 (-2.59, 3.82)	0.71	-1.64 (-4.87, 1.59)	0.32
MBP	-8.99 (-24.0, 8.93)	0.30	-0.99 (-15.4, 13.4)	0.89	1.66 (-4.34, 7.66)	0.59	0.96 (-3.24, 5.17)	0.65	-0.06 (-4.17, 4.05)	0.98
MiBP	-11.0 (-27.9, 9.79)	0.27	-1.67 (-13.8, 10.5)	0.79	3.00 (-2.28, 8.27)	0.26	2.43 (-1.17, 6.04)	0.18	0.04 (-3.55, 3.64)	0.98
MEP	3.82 (-11.8, 22.1)	0.65	-3.68 (-10.9, 3.54)	0.32	2.36 (-0.76, 5.49)	0.14	0.51 (-1.59, 2.61)	0.63	1.18 (-0.94, 3.29)	0.27
MCPP	-22.0 (-31.9, -10.6)	0.00*	14.7 (3.41, 26.0)	0.01*	2.14 (-2.78, 7.05)	0.39	-0.24 (-3.57, 3.08)	0.89	2.14 (-1.16, 5.45)	0.20
**Visit 2 : median 18 weeks of gestation**										
	**ln-TSH**		**Free T4**		**Total T3**		**Total T4**		**T3/T4 Ratio**	
MEHP	3.77 (-6.10, 14.7)	0.47	4.42 (-2.7, 11.5)	0.22	1.79 (-1.56, 5.15)	0.29	1.81 (-0.52, 4.14)	0.13	-0.43 (-3.56, 2.69)	0.79
MEHHP	6.44 (-4.38, 18.5)	0.26	1.36 (-6.23, 8.94)	0.73	4.45 (0.90, 7.99)	0.01*	1.31 (-1.16, 3.77)	0.30	3.25 (-0.05, 6.55)	0.05
MEOHP	8.55 (-2.69, 21.1)	0.14	1.53 (-6.30, 9.37)	0.70	4.31 (0.65, 7.97)	0.02*	2.39 (-0.16, 4.93)	0.07	1.75 (-1.68, 5.18)	0.32
MECPP	11.3 (-0.03, 24.0)	0.05	0.89 (-6.50, 8.28)	0.81	3.41 (-0.05, 6.87)	0.05	3.27 (0.87, 5.67)	0.01*	0.01 (-3.24, 3.26)	0.99
ΣDEHP	9.93 (-1.62, 22.8)	0.09	1.79 (-6.54, 10.1)	0.67	4.11 (0.20, 8.02)	0.04*	2.89 (0.18, 5.60)	0.04*	1.04 (-2.63, 4.70)	0.58
MBzP	-12.6 (-23.5, -0.05)	0.049*	1.63 (-6.94, 10.2)	0.71	3.91 (-0.08, 7.90)	0.06	5.18 (2.42, 7.94)	0.00*	-0.6 (-4.32, 3.12)	0.75
MBP	-12.6 (-23.3, -0.37)	0.04*	9.62 (-1.61, 20.9)	0.09	3.42 (-1.60, 8.44)	0.18	2.49 (-1.37, 6.35)	0.21	1.65 (-3.27, 6.58)	0.51
MiBP	-15.1 (-26.9, -1.51)	0.03*	1.77 (-8.43, 12.0)	0.73	3.05 (-1.57, 7.68)	0.19	4.18 (0.68, 7.68)	0.02*	1.29 (-3.26, 5.85)	0.58
MEP	-4.99 (-18.0, 5.90)	0.28	0.95 (-5.32, 7.22)	0.77	1.44 (-1.46, 4.34)	0.33	0.89 (-1.17, 2.95)	0.40	1.54 (-1.16, 4.25)	0.26
MCPP	-2.65 (-12.7, 8.56)	0.63	6.34 (-5.39, 18.1)	0.29	3.05 (-2.38, 8.48)	0.27	-0.25 (-4.12, 3.63)	0.91	3.44 (-1.66, 8.55)	0.19
**Visit 3 : median 26 weeks of gestation**										
	**ln-TSH**		**Free T4**		**Total T3**		**Total T4**		**T3/T4 Ratio**	
MEHP	-6.31 (-13.9, 1.93)	0.13	-2.48 (-16.6, 11.7)	0.73	-0.13 (-4.02, 3.75)	0.95	0.22 (-2.83, 3.28)	0.89	0.65 (-3.37, 4.67)	0.75
MEHHP	-1.14 (-9.30, 7.74)	0.79	-7.35 (-20.8, 6.14)	0.28	-0.07 (-3.73, 3.59)	0.97	-0.17 (-3.04, 2.70)	0.91	0.82 (-2.97, 4.61)	0.67
MEOHP	-2.58 (-10.5, 6.08)	0.55	-4.18 (-18.8, 10.4)	0.57	0.65 (-3.28, 4.58)	0.75	1.46 (-1.64, 4.57)	0.35	0.46 (-3.62, 4.53)	0.83
MECPP	-2.92 (-11.1, 5.97)	0.51	-3.27 (-17.4, 10.9)	0.65	1.27 (-2.58, 5.13)	0.52	2.67 (-0.30, 5.63)	0.08	0.59 (-3.41, 4.58)	0.77
ΣDEHP	-2.91 (-10.8, 5.73)	0.50	-5.44 (-21.6, 10.7)	0.51	0.92 (-3.48, 5.33)	0.68	2.08 (-1.35, 5.51)	0.23	0.74 (-3.82, 5.30)	0.75
MBzP	-8.68 (-18.2, 1.88)	0.10	-2.26 (-18.3, 13.8)	0.78	1.08 (-3.31, 5.47)	0.63	3.75 (0.36, 7.14)	0.03*	-2.69 (-7.23, 1.85)	0.24
MBP	-6.71 (-15.4, 2.81)	0.16	-0.02 (-21.1, 21.1)	1.00	1.42 (-4.07, 6.90)	0.61	0.50 (-3.81, 4.80)	0.82	0.76 (-4.93, 6.45)	0.79

	ln-TSH		Free T4		Total T3		Total T4		T3/T4 Ratio	
MiBP	-8.29 (-17.5, 1.91)	0.11	-2.98 (-21.0, 15.0)	0.74	-1.53 (-6.41, 3.36)	0.54	0.86 (-2.89, 4.60)	0.65	-0.27 (-5.35, 4.82)	0.92
MEP	-4.11 (-12.3, 4.85)	0.36	-2.07 (-12.5, 8.33)	0.70	2.12 (-0.56, 4.81)	0.12	0.74 (-1.38, 2.86)	0.49	1.83 (-0.96, 4.62)	0.20
MCPP	-6.92 (-14.5, 1.31)	0.10	-9.80 (-31.8, 12.2)	0.38	0.74 (-5.19, 6.67)	0.81	1.24 (-3.31, 5.78)	0.59	0.46 (-5.70, 6.62)	0.88
**Visit 4 : median 35 weeks of gestation**										
	**ln-TSH**		**Free T4**		**Total T3**		**Total T4**		**T3/T4 Ratio**	
MEHP	-6.31 (-14.7, 2.86)	0.17	2.98 (-2.84, 8.80)	0.31	0.31 (-4.31, 4.93)	0.90	4.03 (1.19, 6.88)	0.01*	-6.18 (-10.9, -1.47)	0.01*
MEHHP	0.17 (-9.36, 10.7)	0.97	2.95 (-3.32, 9.21)	0.36	1.20 (-3.74, 6.13)	0.63	1.90 (-1.20, 4.99)	0.23	-4.30 (-9.39, 0.78)	0.10
MEOHP	-1.12 (-10.9, 9.78)	0.83	3.59 (3.00, 10.17)	0.28	1.34 (-3.81, 6.49)	0.61	3.01 (-0.23, 6.25)	0.07	-5.76 (-11.0, -0.48)	0.03*
MECPP	-0.74 (-11.5, 11.4)	0.90	4.32 (-2.94, 11.6)	0.24	1.68 (-3.86, 7.23)	0.55	4.23 (0.65, 7.80)	0.02*	-5.37 (-11.1, 0.33)	0.06
ΣDEHP	-2.03 (-12.3, 9.49)	0.72	4.43 (-2.55, 11.4)	0.21	1.71 (-3.75, 7.17)	0.54	3.99 (0.56, 7.42)	0.02*	-5.88 (-11.5, -0.28)	0.04*
MBzP	5.29 (-5.63, 17.5)	0.35	8.26 (1.34, 15.2)	0.02*	-2.84 (-8.03, 2.35)	0.28	0.42 (-3.03, 3.87)	0.81	-6.09 (-11.4, -0.77)	0.03*
MBP	-1.29 (-12.3, 11.1)	0.83	3.42 (-4.07, 10.9)	0.37	-0.51 (-6.00, 4.98)	0.85	0.02 (-3.67, 3.71)	0.99	-3.60 (-9.26, 2.06)	0.21
MiBP	-2.44 (-13.4, 9.96)	0.68	3.45 (-4.35, 11.2)	0.39	-1.23 (-6.94, 4.48)	0.67	0.37 (-3.56, 4.29)	0.85	-1.58 (-7.74, 4.58)	0.61
MEP	-2.04 (-11.8, 8.79)	0.70	2.97 (-3.82, 9.77)	0.39	3.34 (-1.59, 8.27)	0.18	0.61 (-2.78, 3.99)	0.73	3.01 (-2.19, 8.22)	0.26
MCPP	-5.15 (-14.3, 5.01)	0.31	8.55 (2.05, 15.1)	0.01*	1.64 (-3.11, 6.38)	0.50	0.51 (-2.74, 3.75)	0.76	-1.26 (-6.18, 3.66)	0.61

Linear regression models adjusted for urinary specific gravity, gestational age at time of sample collection, maternal age at enrollment, body mass index (BMI) at time of sample collection, and health insurance provider.

*p<0.05
